# Supplementary material for: Longitudinal plasma proteomics reveals biomarkers of alveolar-capillary barrier disruption in critically ill COVID-19 patients
Source: Nat Commun. 2024 Jan 25;15:744. doi: 10.1038/s41467-024-44986-w (PMC10811341; doi:10.1038/s41467-024-44986-w)
Supplement: Supplementary file 1 — Supplementary Information [file 41467_2024_44986_MOESM1_ESM.pdf]

Supplementary Appendix to: *Longitudinal plasma proteomics reveals biomarkers of alveolar-capillary barrier disruption in critically ill COVID-19 patients*

Erik Duijvelaar MD, Jack Gisby MSc, James E Peters PhD, Harm Jan Bogaard PhD, Jurjan Aman PhD

Content

TABLE S1. CHARACTERISTICS OF INCLUDED PATIENTS (≥1 PLASMA SAMPLE AVAILABLE) VERSUS EXCLUDED PATIENTS ____ 2

TABLE S2. FACTORS ASSOCIATED WITH FLAGGING OF A SAMPLE ____ 4

TABLE S3. PERCENTAGE OF VARIANCE EXPLAINED BY EACH VARIABLE OF INTEREST ____ 5

TABLE S4. CHARACTERISTICS OF PATIENTS FROM THE VUMC HOSPITAL ____ 6

TABLE S5. CHARACTERISTICS OF PATIENTS TREATED WITH PLACEBO AND IMATINIB ____ 7

TABLE S6. OVERLAP BETWEEN PROTEINS AND GENES AFFECTED BY IMATINIB IN HUMAN PLASMA AND IN THE LUNGS OF SYRIAN HAMSTERS ____ 8

FIGURE S1. PATIENT AND SAMPLE SELECTION ____ 9

FIGURE S2. QUALITY CONTROL: RELATIVE LOG EXPRESSION PLOTS ____ 10

FIGURE S3. QUALITY CONTROL: PRINCIPAL COMPONENT ANALYSIS ____ 11

FIGURE S4. VARIANCE EXPLAINED BY EACH VARIABLE OF INTEREST ____ 12

FIGURE S5. RELATIVE PROTEIN ABUNDANCE OF THE 50 MOST SIGNIFICANTLY DIFFERENT PROTEINS CRITICAL AND NON-CRITICAL DISEASE AT ADMISSION ____ 13

FIGURE S6. RELATIVE PROTEIN ABUNDANCE OF THE 50 MOST SIGNIFICANTLY DIFFERENT PROTEINS CRITICAL AND NON-CRITICAL DISEASE AFTER 3 DAYS ____ 14

FIGURE S7. DYSREGULATED PATHWAYS IN CRITICAL DISEASE AT HOSPITAL ADMISSION ____ 15

FIGURE S8. PROTEINS WITH A DIFFERENT CHANGE OVER TIME IN PATIENTS WHO DEVELOPED A CRITICAL DISEASE COURSE ____ 16

FIGURE S9. PROTEINS ASSOCIATED WITH TIME TO INTUBATION OR DEATH ____ 17

FIGURE S10. PREDICTING CRITICAL ILLNESS DEVELOPMENT, DIFFERENT VALIDATION COHORTS ____ 18

FIGURE S11. PROTEIN ABUNDANCE IN PATIENTS RANDOMISED TO IMATINIB OR PLACEBO AT HOSPITAL ADMISSION ____ 19

FIGURE S12. RELATIVE PROTEINS ABUNDANCE IN PATIENTS TREATED WITH IMATINIB AND PLACEBO AFTER 3 DAYS ____ 20

FIGURE S13 EFFECT OF IMATINIB ON THE TRANSCRIPTOME OF SYRIAN HAMSTER LUNGS ____ 21

FIGURE S14 PATHWAYS AFFECTED BY IMATINIB IN THE TRANSCRIPTOME OF SYRIAN HAMSTER LUNGS ____ 22

FIGURE S15 GENOTYPIC ALTERATIONS ACROSS VARIOUS CELL TYPES IN FATAL COVID-19 ____ 23

Table S1. Characteristics of included patients (≥1 plasma sample available) versus excluded patients

	Excluded in secondary analysis	Included in secondary analysis	p-value
**Patients, n**	67	318	
**Demographic variables**			
**Age, mean (SD)**	63.1 (11.9)	64.3 (12.3)	0.437
**Male sex, n (%)**	42 (62.7)	222 (69.8)	0.319
**BMI, mean (SD)**	30.2 (5.9)	29.2 (5.3)	0.196
**History of smoking or current smoker, n (%)**	29 (48.3)	124 (40.4)	0.318
**Comorbid diabetes mellitus, n (%)**	11 (16.4)	89 (28.0)	0.070
**Comorbid cardiovascular disease, n (%)**	13 (19.4)	70 (22.0)	0.758
**Comorbid atrial fibrillation, n (%)**	2 (3.0)	30 (9.4)	0.135
**Comorbid hypertension, n (%)**	25 (37.3)	120 (37.7)	1.000
**Treatment, n (%)**			
**Imatinib**	35 (52.2)	162 (50.9)	0.953
**Dexamethasone**	49 (73.1)	227 (71.4)	0.889
**Remdesivir**	18 (26.9)	62 (19.5)	0.236
**Chloroquine**	4 (6.0)	32 (10.1)	0.415
**Laboratory findings at admission**			
**Hemoglobin (mmol/L), mean (SD)**	8.4 (0.9)	8.5 (0.9)	0.640
**Leukocytes (×10^9/L), mean (SD)**	8.3 (3.3)	8.6 (4.7)	0.646
**Neutrophils (×10^9/L), mean (SD)**	6.8 (3.5)	6.8 (3.5)	0.939
**Lymphocytes (×10^9/L), mean (SD)**	1.0 (0.4)	1.0 (0.5)	0.456
**Thrombocytes (×10^9/L), mean (SD)**	279 (118)	263 (108)	0.303
**eGFR (ml/min/1,73 m2), mean (SD)**	79.0 (16.3)	77.8 (17.8)	0.628
**CRP (mg/L), mean (SD)**	113 (98)	111 (76)	0.840
**Hs-cTnT (ng/L), median [IQR]**	7.5 [4.0-16.3]	8.0 [5.0-15.0)	0.868
**NTproBNP (ng/L), median [IQR]**	131 [55, 303]	142 [42, 394]	0.720
**LDH (U/L), mean (SD)**	396 (162)	390 (148)	0.743
**Albumin (g/L), mean (SD)**	40.0 (48.6)	35.2 (5.7)	0.132
**Clinical characteristics at admission**			
**SpO2 (%), mean (SD)**	93.7 (2.8)	94.0 (2.5)	0.382
**FiO2, mean (SD)**	40.0 (24.2)	37.6 (20.4)	0.399
**SpO2/FiO2, mean (SD)**	297 (111)	301 (101)	0.779
**modified WHO ordinal scale, n (%)**			0.063
**Hospitalised, no oxygen supplementation**	1 (1.5)	8 (2.5)	
**Oxygen mask or nasal cannula**	62 (92.5)	293 (92.1)	
**NIV or HFNO**	2 (3.0)	16 (5.0)	
**Invasive ventilation and additional organ support**	0 (0.0)	1 (0.3)	
**Death**	1 (1.5)	0 (0.0)	
**Clinical course**			
**length of admission (days), median [IQR]**	6.0 [3.0, 12.5]	7.0 [4.0, 11.0]	0.638
**length of invasive ventilation (days), median [IQR]**	8.0 [7.0, 14.8]	10.0 [4.8, 20.0]	0.912
**Need for ICU admission, n (%)**	13 (19.4)	59 (18.6)	1.000
**Need for invasive ventilation, n (%)**	12 (17.9)	44 (13.8)	0.504
**Mortality, n (%)**	9 (13.4)	40 (12.6)	1.000

BMI = body mass index, CRP = C-reactive protein, eGFR = estimated glomerular filtration rate, FiO2 = fraction of inspired oxygen, HFNO = high flow nasal oxygen, ICU = intensive care unit, IQR = interquartile range, LDH = Lactate dehydrogenase, NIV = non-invasive ventilation, SD = standard deviation, SpO2 = Peripheral oxygen saturation, WHO = World Health Organization. For normally distributed numeric variables (presented as mean (SD)),

two-sided unpaired t-tests were used; for non-normally distributed numeric variables (median [IQR]), two-sided Mann-Whitney U tests were applied. Chi-square tests were used for categorical variables (n (%)).

Table S2. Factors associated with flagging of a sample

Number of samples: 535	**Odds ratio**	**95% CI**	**p-value**
**Demographic variables - n (%)**			
Sex (Male)	0.95	0.40, 2.27	0.92
Age (years)	1.02	0.98, 1.06	0.34
Comorbid obesity (BMI >30 kg/m²)	1.29	0.56, 2.95	0.55
Comorbid diabetes	0.36	0.13, 1.01	0.053
**Clinical variables**			
Need for invasive ventilation	0.98	0.17, 5.74	0.99
Critical disease	0.62	0.12, 3.10	0.56
**Laboratory findings**			
eGFR (ml/min/1,73 m2)	1.00	0.97, 1.03	0.99
CRP (mg/L)	1.00	1.00, 1.01	0.73
**Medication use**			
Imatinib (reference = placebo)	0.76	0.35, 1.64	0.48
Therapeutic anticoagulation	0.81	0.20, 3.41	0.78
Platelet aggregation inhibitor	0.74	0.28, 1.99	0.56
Dexamethasone	0.78	0.27, 2.26	0.64
Remdesivir	2.24	0.81, 6.23	0.12
**Technical issues**			
Clogged/low volume during sample processing	17.9	7.4, 43.2	1.58e-10

BMI = body mass index, CRP = C-reactive protein, CI= confidence interval, eGFR = estimated glomerular filtration rate. Multivariate logistic regression was applied to determine the odds ratios of each variable for flagging of a sample.

Table S3. Percentage of variance explained by each variable of interest

**Variable**	**Variance explained (%)**
Hospital site (factor)	6.56
Flagging of samples* (dichotomous)	6.47
Critical disease (dichotomous)	1.51
Time (dichotomous)	1.07
Sex (dichotomous)	0.59
Comorbid diabetes mellitus (dichotomous)	0.59
Obesity (dichotomous)	0.58
Comorbid cardiovascular disease (dichotomous)	0.47
Treatment (dichotomous)	0.29

* Hybridization control, intraplate median signal, and median signal normalization were performed to remove systematic biases. Normalization ratios that exceed the interval of 0.40 – 2.50 were flagged

Table S4. Characteristics of patients from the VUmc hospital

	All hospitals except VUmc hospital	VUmc hospital	p-value
**Patients, n**	256	62	
**Demographic variables**			
**Age, mean (SD)**	64.1 (12.5)	65.4 (11.7)	0.449
**Male sex, n (%)**	176 (68.8)	46 (74.2)	0.494
**BMI, mean (SD)**	29.2 (5.3)	29.2 (5.2)	0.959
**History of smoking or current smoker, n (%)**	95 (38.6)	29 (47.5)	0.260
**Comorbid diabetes mellitus, n (%)**	71 (27.7)	18 (29.0)	0.963
**Comorbid cardiovascular disease, n (%)**	61 (23.8)	9 (14.5)	0.156
**Comorbid atrial fibrillation, n (%)**	19 (7.4)	11 (17.7)	0.024
**Comorbid hypertension, n (%)**	94 (36.7)	26 (41.9)	0.539
**Treatment, n (%)**			
**Imatinib**	131 (52.2)	31 (50.0)	0.981
**Dexamethasone**	184 (71.9)	43 (69.4)	0.812
**Remdesivir**	55 (21.5)	7 (11.3)	0.101
**Chloroquine**	18 (7.0)	14 (22.6)	0.001
**Laboratory findings at admission**			
**Hemoglobin (mmol/L), mean (SD)**	8.4 (0.9)	8.7 (0.9)	0.009
**Leukocytes (×10^9/L), mean (SD)**	8.6 (4.9)	8.8 (3.9)	0.691
**Neutrophils (×10^9/L), mean (SD)**	6.7 (3.5)	7.3 (3.8)	0.181
**Lymphocytes (×10^9/L), mean (SD)**	1.0 (0.5)	0.9 (0.4)	0.073
**Thrombocytes (×10^9/L), mean (SD)**	267 (108)	249 (104)	0.236
**eGFR (ml/min/1,73 m2), mean (SD)**	77.6 (18.5)	78.5 (14.6)	0.746
**CRP (mg/L), mean (SD)**	104.5 (73.3)	134.3 (79.5)	0.006
**Hs-cTnT (ng/L), median [IQR]**	8.0 [5.0, 15.0]	8.0 [6.0, 14.5]	0.802
**NTproBNP (ng/L), median [IQR]**	114 [34, 337]	286 [128, 776]	2.13e-4
**LDH (U/L), mean (SD)**	380 (142)	428 (168)	0.024
**Albumin (g/L), mean (SD)**	35.0 (6.0)	35.9 (3.7)	0.364
**Clinical characteristics at admission**			
**SpO2 (%), mean (SD)**	94.1 (2.5)	93.4 (2.7)	0.050
**FiO2, mean (SD)**	36.3 (19.8)	42.8 (22.4)	0.025
**SpO2/FiO2, mean (SD)**	308 (98)	207 (107)	0.007
**modified WHO ordinal scale, n (%)**			0.016
**Hospitalised, no oxygen supplementation**	8 (3.1)	0 (0.0)	
**Oxygen mask or nasal cannula**	232 (90.6)	61 (98.4)	
**NIV or HFNO**	16 (6.2)	0 (0.0)	
**Invasive ventilation and additional organ support**	0 (0.0)	1 (1.6)	
**Clinical course**			
**length of admission (days), median [IQR]**	7.0 [3.0, 10.0]	7.0 [5.0, 16.0]	0.023
**length of invasive ventilation (days), median [IQR]**	6.5 [3.0, 20.2]	10.5 [7.8, 19.2]	0.074
**Need for ICU admission, n (%)**	41 (16.0)	18 (29.0)	0.029
**Need for invasive ventilation, n (%)**	28 (10.9)	16 (25.8)	0.005
**Mortality, n (%)**	29 (11.3)	11 (17.7)	0.249

BMI = body mass index, CRP = C-reactive protein, eGFR = estimated glomerular filtration rate, FiO2 = fraction of inspired oxygen, HFNO = high flow nasal oxygen, ICU = intensive care unit, IQR = interquartile range, LDH = Lactate dehydrogenase, NIV = non-invasive ventilation, SD = standard deviation, SpO2 = Peripheral oxygen saturation, WHO = World Health Organization. For normally distributed numeric variables (presented as mean (SD)), two-sided unpaired t-tests were used; for non-normally distributed numeric variables (median [IQR]), two-sided Mann-Whitney U tests were applied. Chi-square tests were used for categorical variables (n (%)).

Table S5. Characteristics of patients treated with placebo and imatinib

	Placebo	Imatinib	p-value
**Patients, n**	156	162	
**Demographic variables**			
**Age, mean (SD)**	64.3 (12.1)	64.4 (12.5)	0.906
**Male sex, n (%)**	103 (66.0)	119 (73.5)	0.187
**BMI, mean (SD)**	29.9 (5.3)	28.5 (5.2)	0.024
**History of smoking or current smoker, n (%)**	66 (44.0)	58 (36.9)	0.253
**Comorbid diabetes mellitus, n (%)**	53 (34.0)	36 (22.2)	0.027
**Comorbid cardiovascular disease, n (%)**	43 (27.6)	27 (16.7)	0.027
**Comorbid atrial fibrillation, n (%)**	20 (12.8)	10 (6.2)	0.066
**Comorbid hypertension, n (%)**	65 (41.7)	55 (34.0)	0.192
**Treatment, n (%)**			
**Dexamethasone**	111 (71.2)	116 (71.6)	1.000
**Remdesivir**	32 (20.5)	30 (18.5)	0.759
**Chloroquine**	16 (10.3)	16 (9.9)	1.000
**Laboratory findings at admission**			
**Hemoglobin (mmol/L), mean (SD)**	8.5 (1.0)	8.4 (0.9)	0.563
**Leukocytes (×10^9/L), mean (SD)**	8.8 (5.7)	8.4 (3.4)	0.426
**Neutrophils (×10^9/L), mean (SD)**	6.9 (3.8)	6.7 (3.3)	0.664
**Lymphocytes (×10^9/L), mean (SD)**	1.0 (0.5)	1.0 (0.5)	0.763
**Thrombocytes (×10^9/L), mean (SD)**	260 (106)	267 (110)	0.561
**eGFR (ml/min/1,73 m2), mean (SD)**	76.2 (18.7)	79.3 (16.9)	0.128
**CRP (mg/L), mean (SD)**	110 (83)	112 (68)	0.863
**Hs-cTnT (ng/L), median [IQR]**	9.0 [6.0, 17.0]	8.0 [4.0, 12.5]	0.045
**NTproBNP (ng/L), median [IQR]**	124 [43, 362]	155 [43, 459]	0.654
**LDH (U/L), mean (SD)**	412 (161)	369 (132)	0.011
**Albumin (g/L), mean (SD)**	35.4 (5.5)	34.9 (5.9)	0.502
**Clinical characteristics at admission**			
**SpO2 (%), mean (SD)**	93.7 (2.5)	94.3 (2.6)	0.023
**FiO2, mean (SD)**	39.0 (22.2)	36.2 (18.5)	0.221
**SpO2/FiO2, mean (SD)**	3.0 (1.1)	3.1 (1.0)	0.356
**modified WHO ordinal scale, n (%)**			0.788
**Hospitalised, no oxygen supplementation**	4 (2.6)	4 (2.5)	
**Oxygen mask or nasal cannula**	143 (91.7)	150 (92.6)	
**NIV or HFNO**	8 (5.1)	8 (4.9)	
**Invasive ventilation and additional organ support**	1 (0.6)	0 (0.0)	
**Clinical course**			
**length of admission (days), median [IQR]**	7.0 [4.0, 12.0]	7.0 [4.0, 10.8]	0.894
**length of invasive ventilation (days), median [IQR]**	14.0 [6.2, 21.5]	6.5 [3.0, 14.8]	0.037
**Need for ICU admission, n (%)**	29 (18.6)	30 (18.5)	1.000
**Need for invasive ventilation, n (%)**	22 (14.1)	22 (13.6)	1.000
**Mortality, n (%)**	28 (17.9)	12 (7.4)	0.008

BMI = body mass index, CRP = C-reactive protein, eGFR = estimated glomerular filtration rate, FiO2 = fraction of inspired oxygen, HFNO = high flow nasal oxygen, ICU = intensive care unit, IQR = interquartile range, LDH = Lactate dehydrogenase, NIV = non-invasive ventilation, SD = standard deviation, SpO2 = Peripheral oxygen saturation, WHO = World Health Organization. For normally distributed numeric variables (presented as mean (SD)), two-sided unpaired t-tests were used; for non-normally distributed numeric variables (median [IQR]), two-sided Mann-Whitney U tests were applied. Chi-square tests were used for categorical variables (n (%)).

Table S6. Overlap between proteins and genes affected by imatinib in human plasma and in the lungs of Syrian hamsters

Protein	GeneID	Change in human plasma	Change in hamster's lungs
Calcipressin-3	RCAN3	Decrease	Increase
Four-jointed box protein 1	FJX1	Decrease	Decrease
Interleukin-6	IL6	Decrease	Decrease
Peptidyl-prolyl cis-trans isomerase C	PPIC	Decrease	Decrease
Transforming growth factor beta-3	TGFBR3	Increase	Decrease
Urokinase-type plasminogen activator	PLAU	Decrease	Decrease

Figure S1 Patient and sample selection

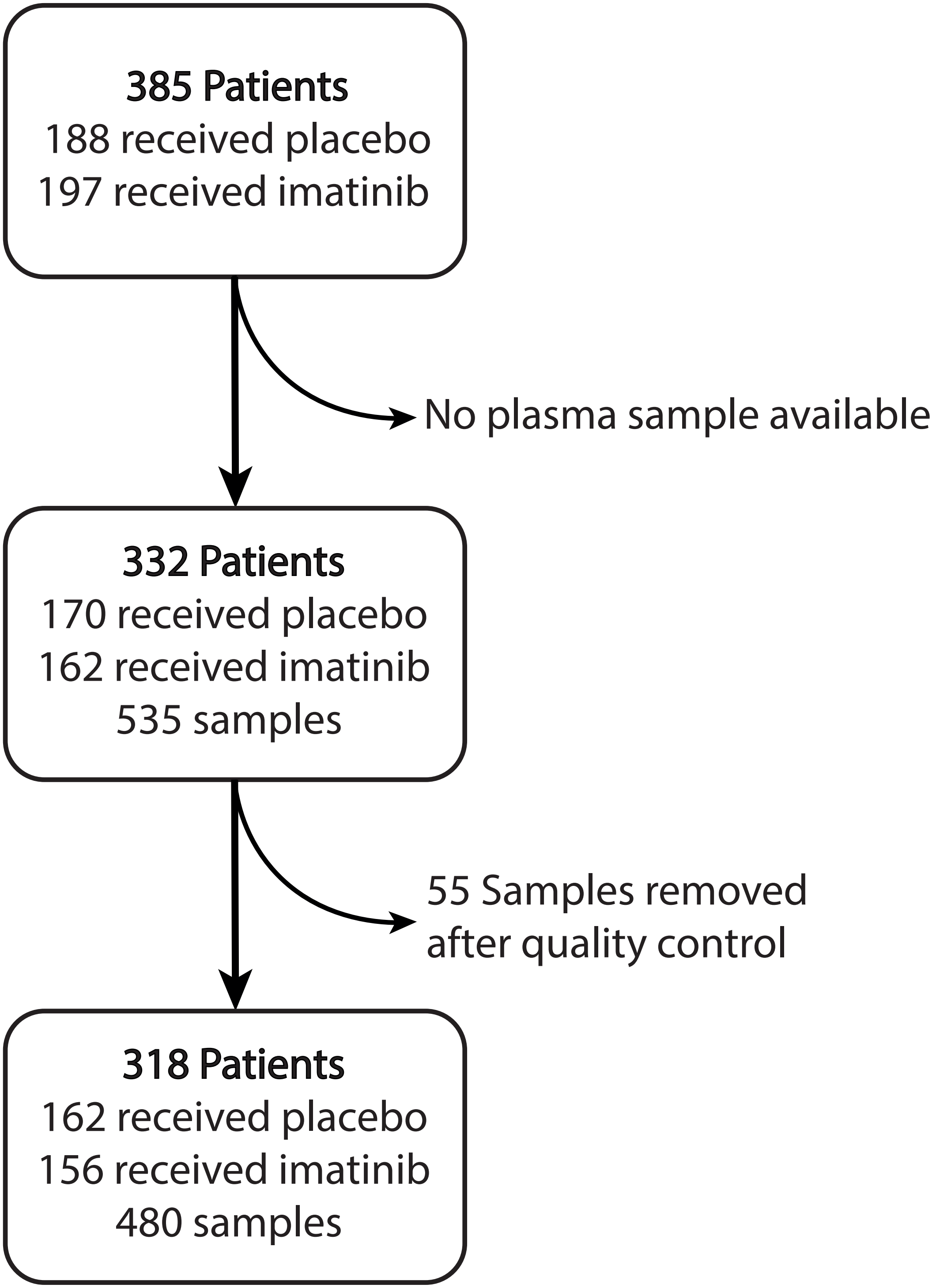

Figure S2 Quality control: relative log expression plots

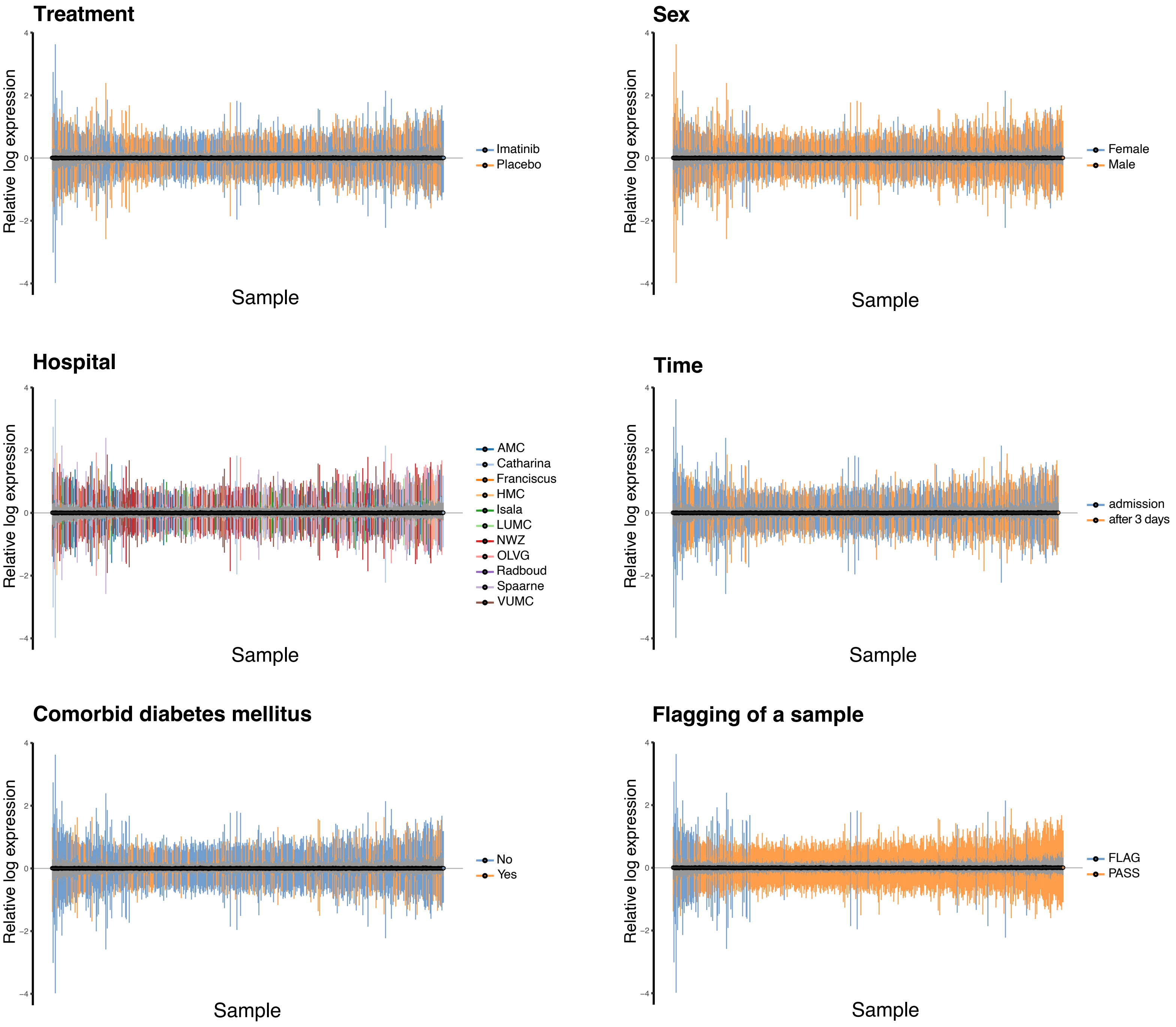

Plots were created using the plotRLE function from the Bioconductor scater package. n=535 samples from 332 individuals

Figure S3 Quality control: Principal component analysis

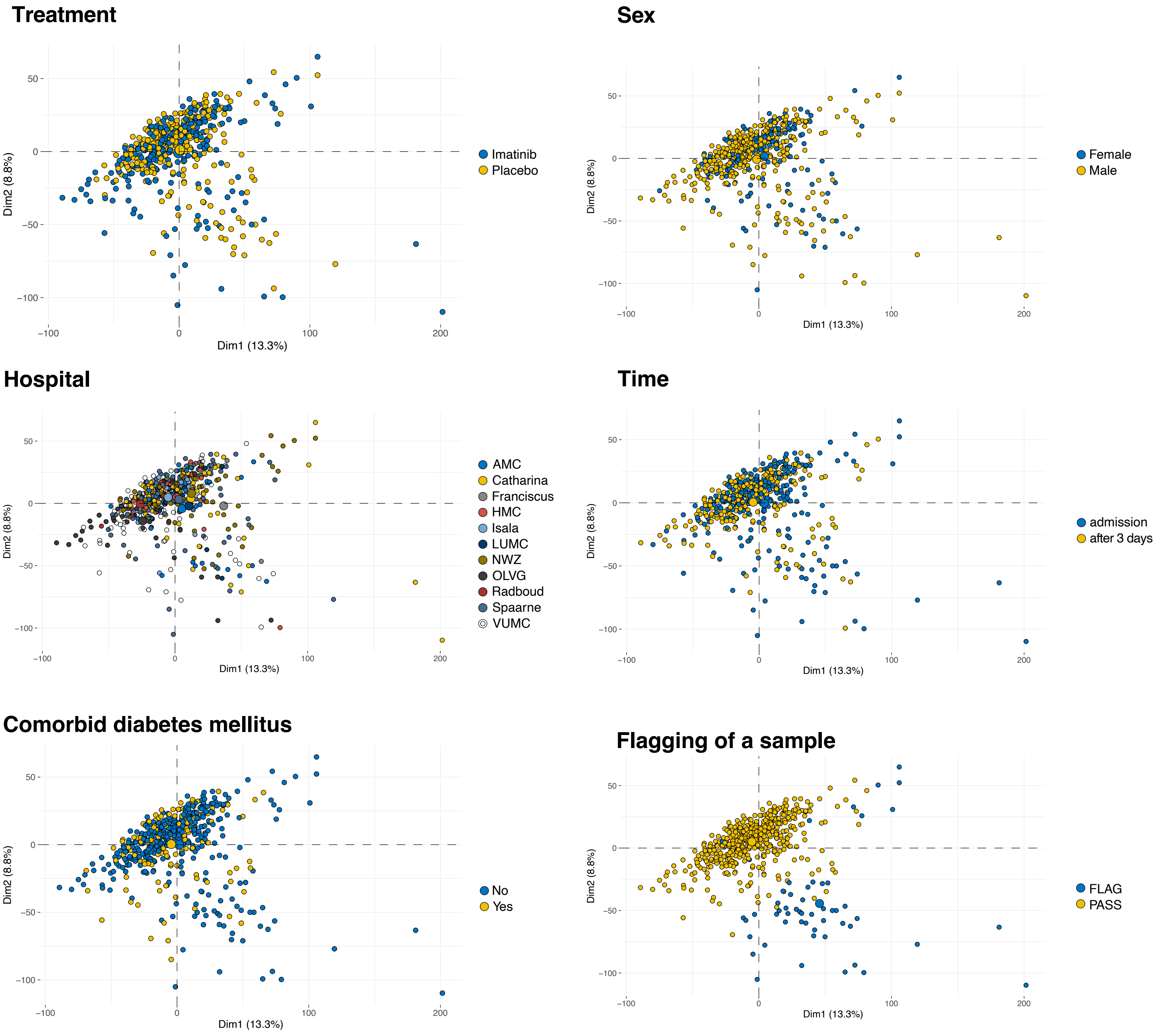

Principal component analyses were performed with the prcomp function from the stats package. n=535 samples from 332 individuals

Figure S4 Quality control: Variance explained by each variable of interest

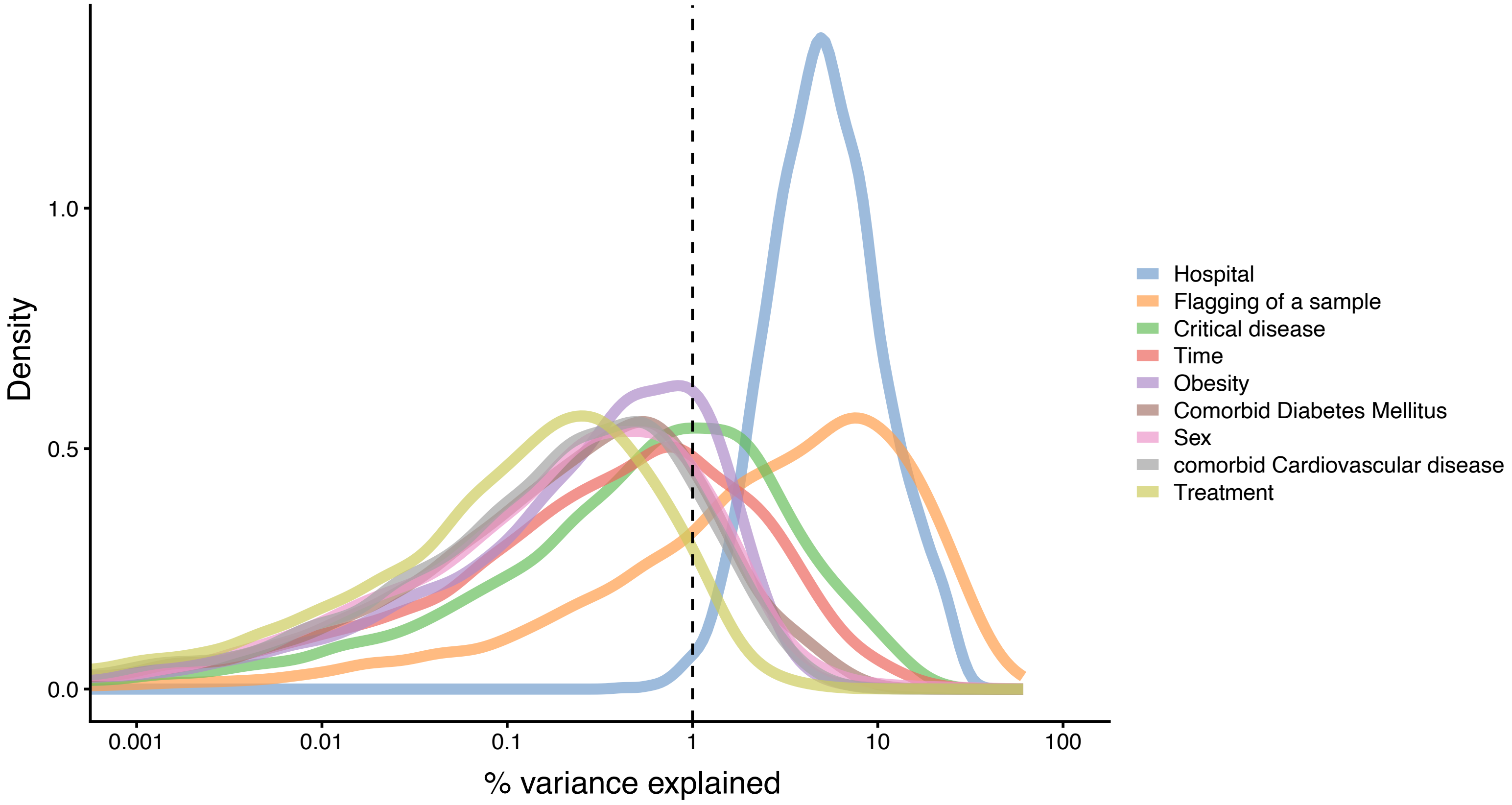

Explained variance was calculated using the getVarianceExplained function from the scater package. n=535 samples from 332 individuals

Figure S5 Relative protein abundance of the 50 most significantly different proteins in critical and non-critical disease at admission

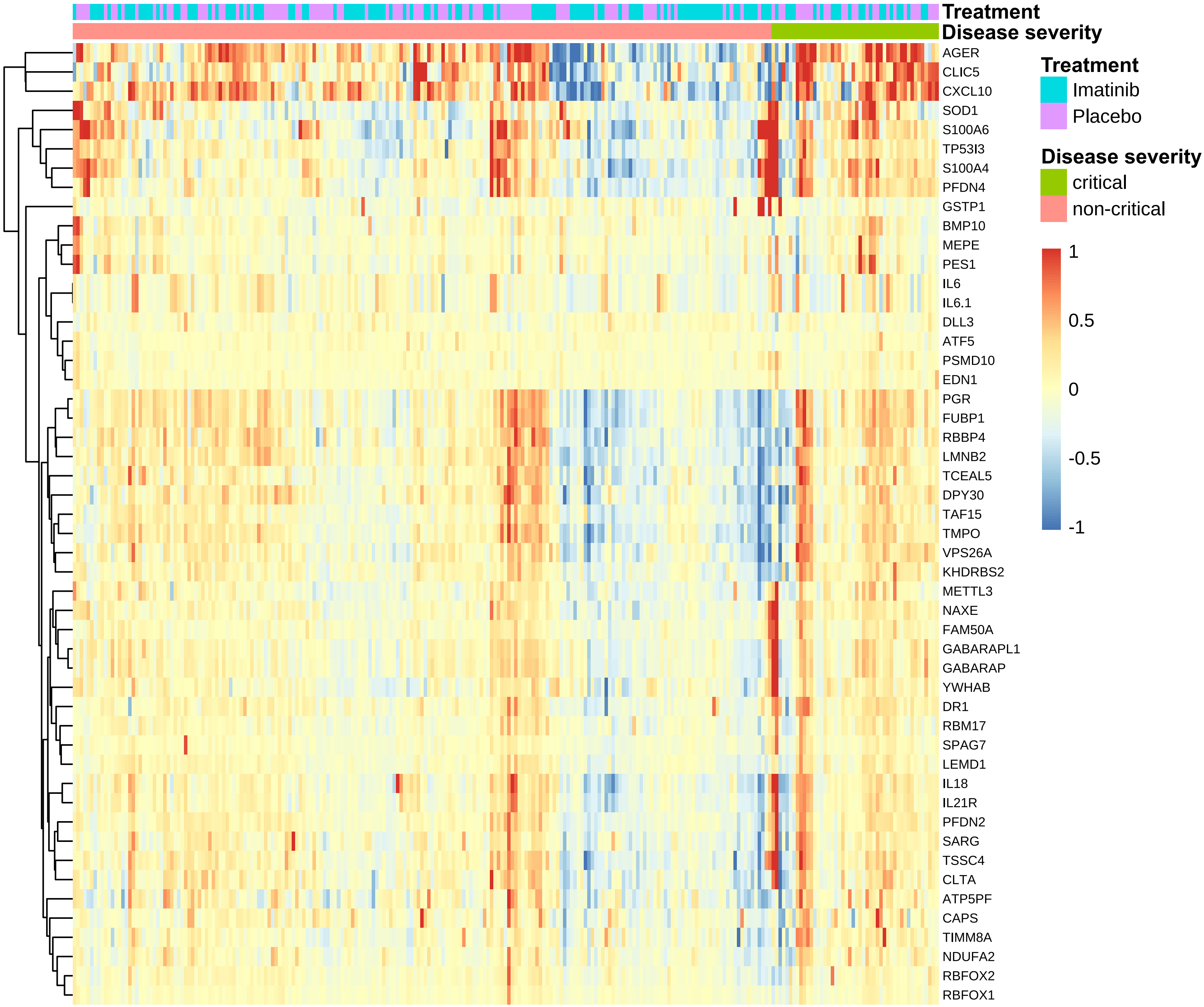

The heatmap was created using the pheatmap function from the pheatmap package on the output from linear mixed models. n= 249 samples/individuals

Figure S6 Relative protein abundance of the 50 most significantly different proteins in critical and non-critical disease after 3 days

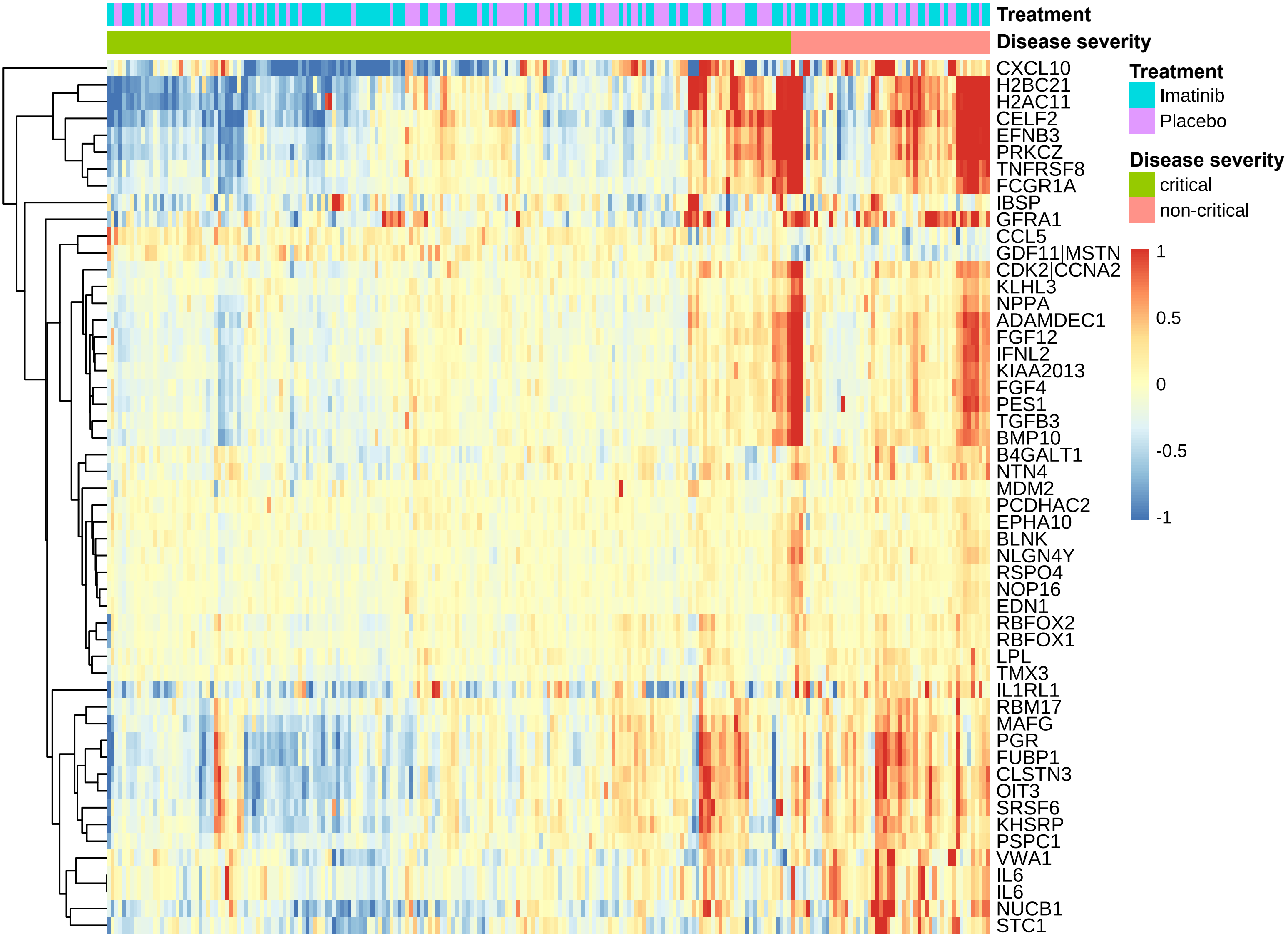

The heatmap was created using the pheatmap function from the pheatmap package on the output from linear mixed models. n= 231 samples/individuals

Figure S7. Dysregulated pathways in critical disease at hospital admission

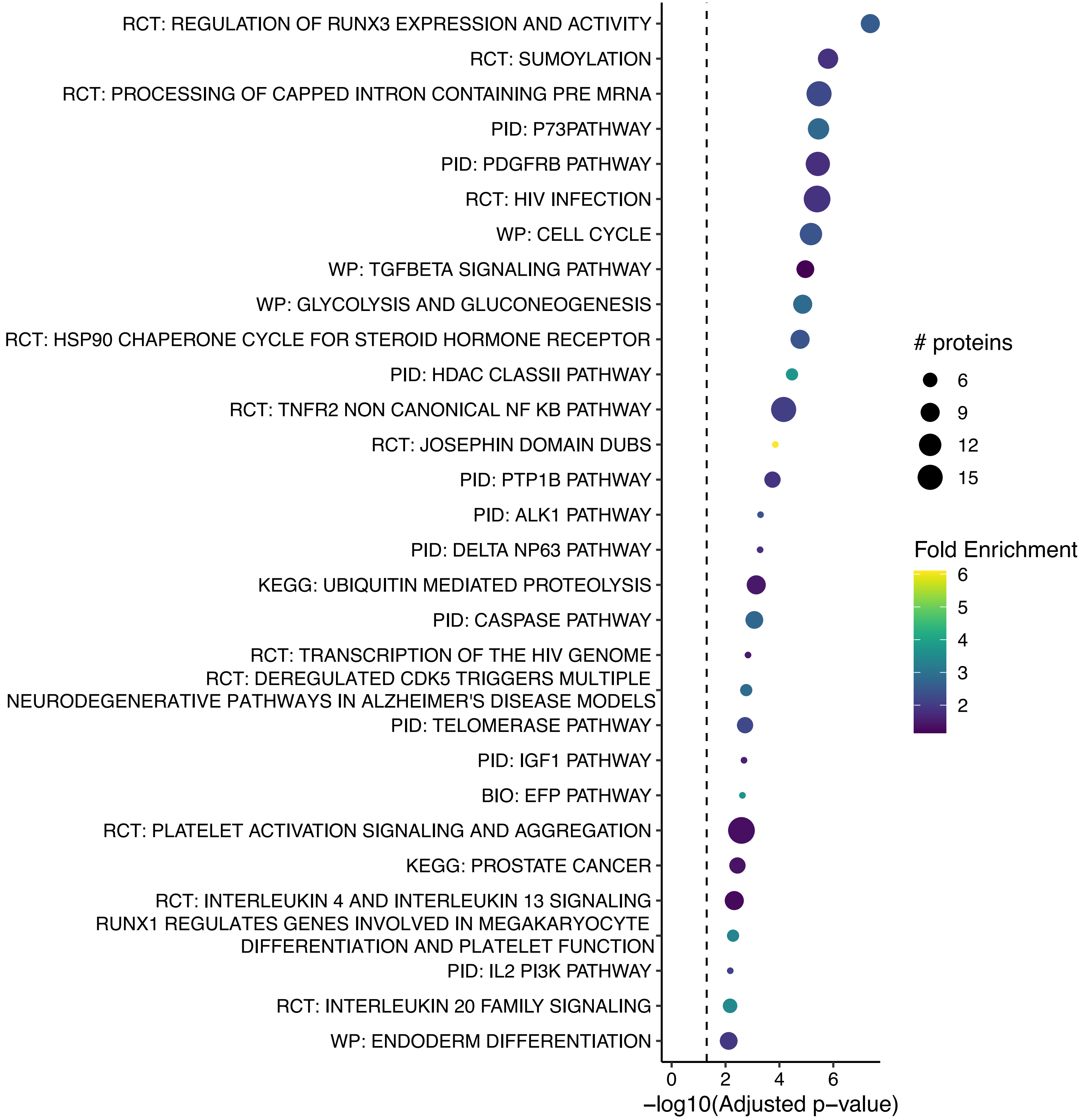

Dotplot of enriched pathways at hospital admission identified by PathfindR (follow-up). The 30 most significant clusters are represented by the most significant pathway term of each cluster. Dot size indicates the number of proteins, colors represent fold enrichment. Terms are ordered by significance. BIO = Biocarta, KEGG = Kyoto Encyclopedia of Genes and Genomes, PID = Pathway Interaction Database, RCT = Reactome, WP = Wikipathways. n= 249 samples/individuals

Figure S8 Proteins with a different change over time in patients who developed a critical disease course

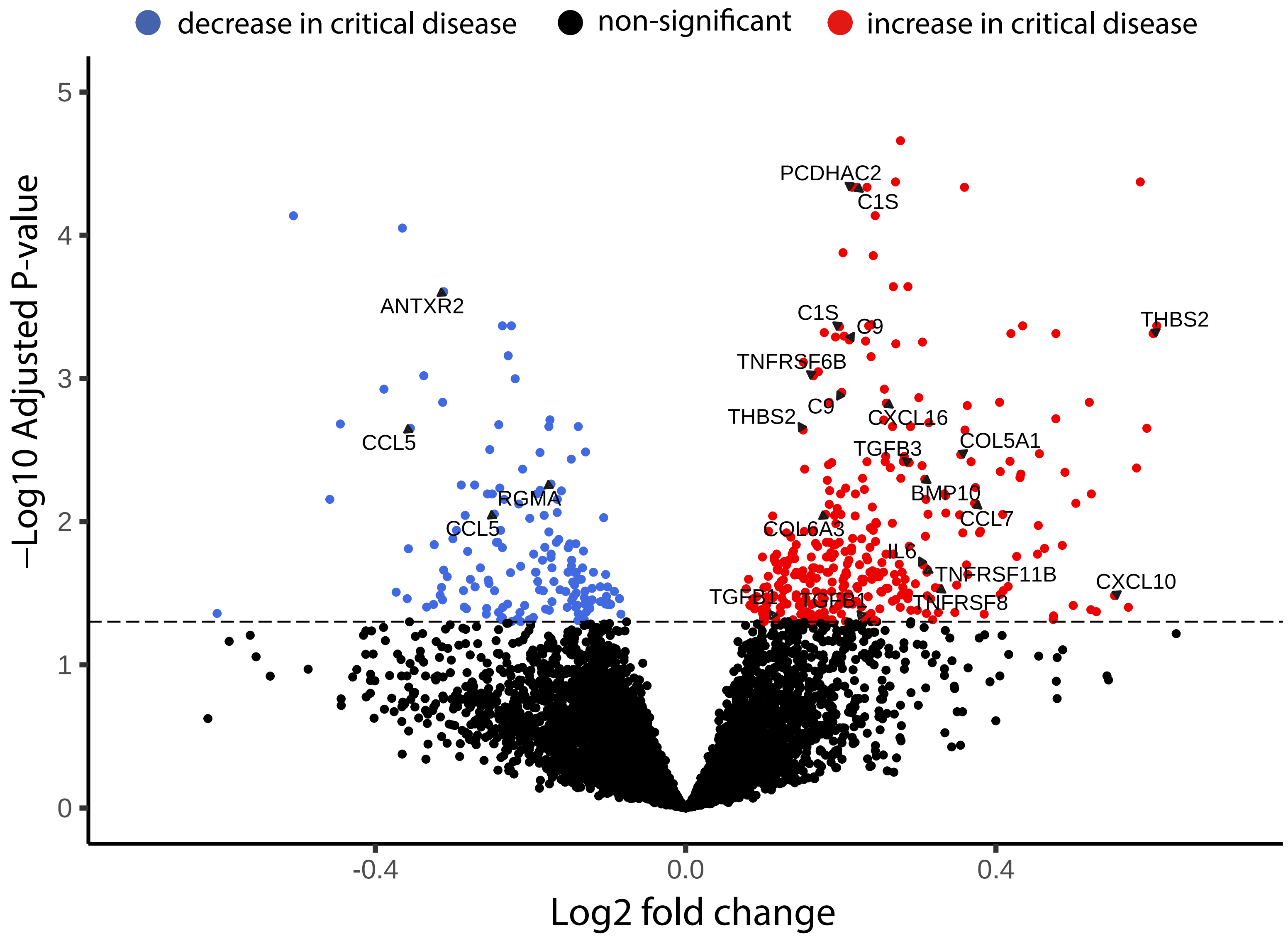

Volcano plot of proteins with different temporal trajectories in critical versus non-critical patients. Log2 fold change and significance levels were calculated using linear mixed models. P-values were adjusted using the Benjamini-Hochberg method. n = 480 samples from 318 individuals

Figure S9 Proteins associated with time to intubation or death

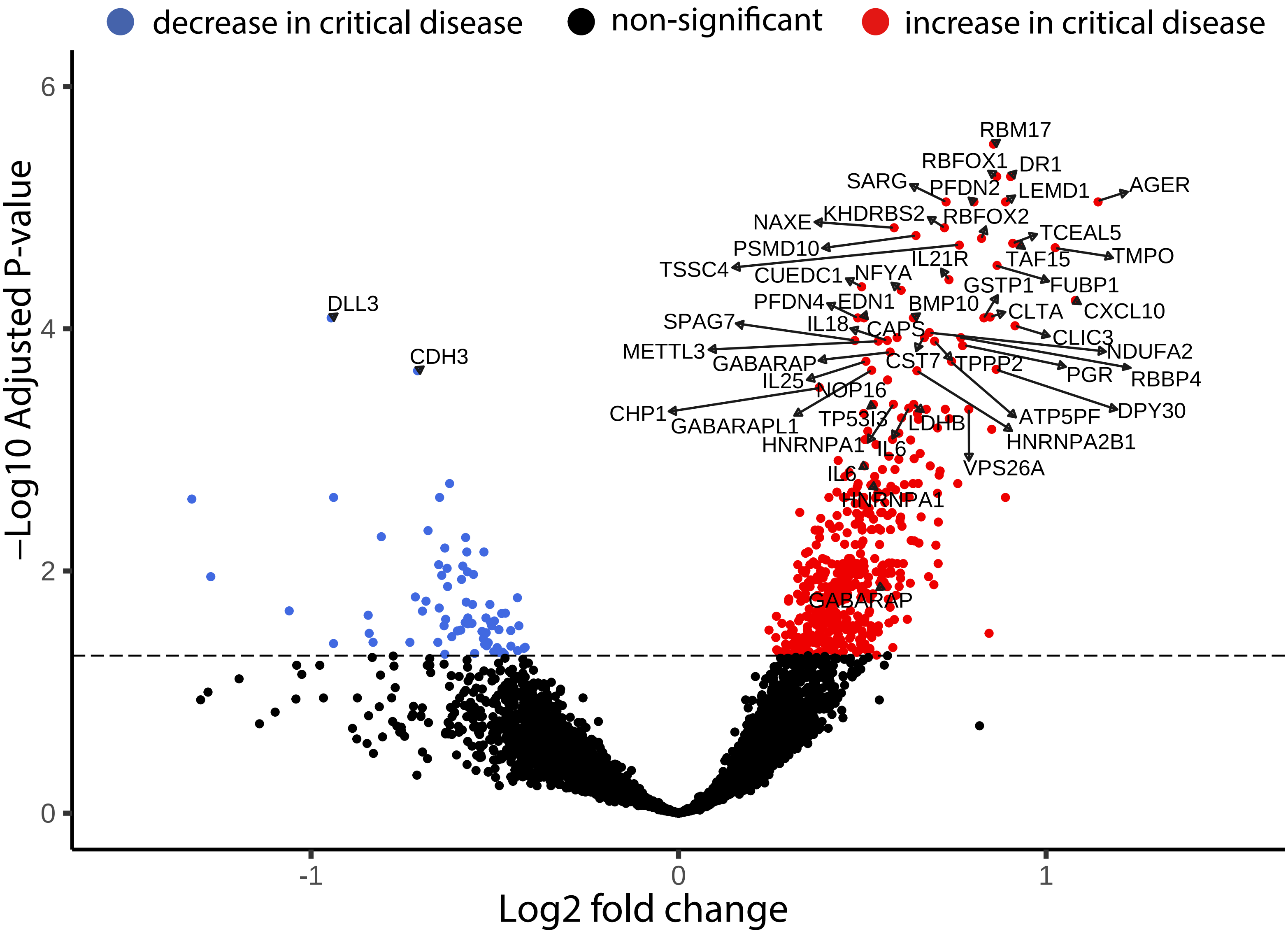

Volcano plot of proteins with a significant association with time to critical illness development. Log2 fold change and significance levels were calculated using linear mixed models. P-values were adjusted using the Benjamini-Hochberg method. n = 249 samples/individuals

Figure S10 Predicting critical illness development, different validation cohorts

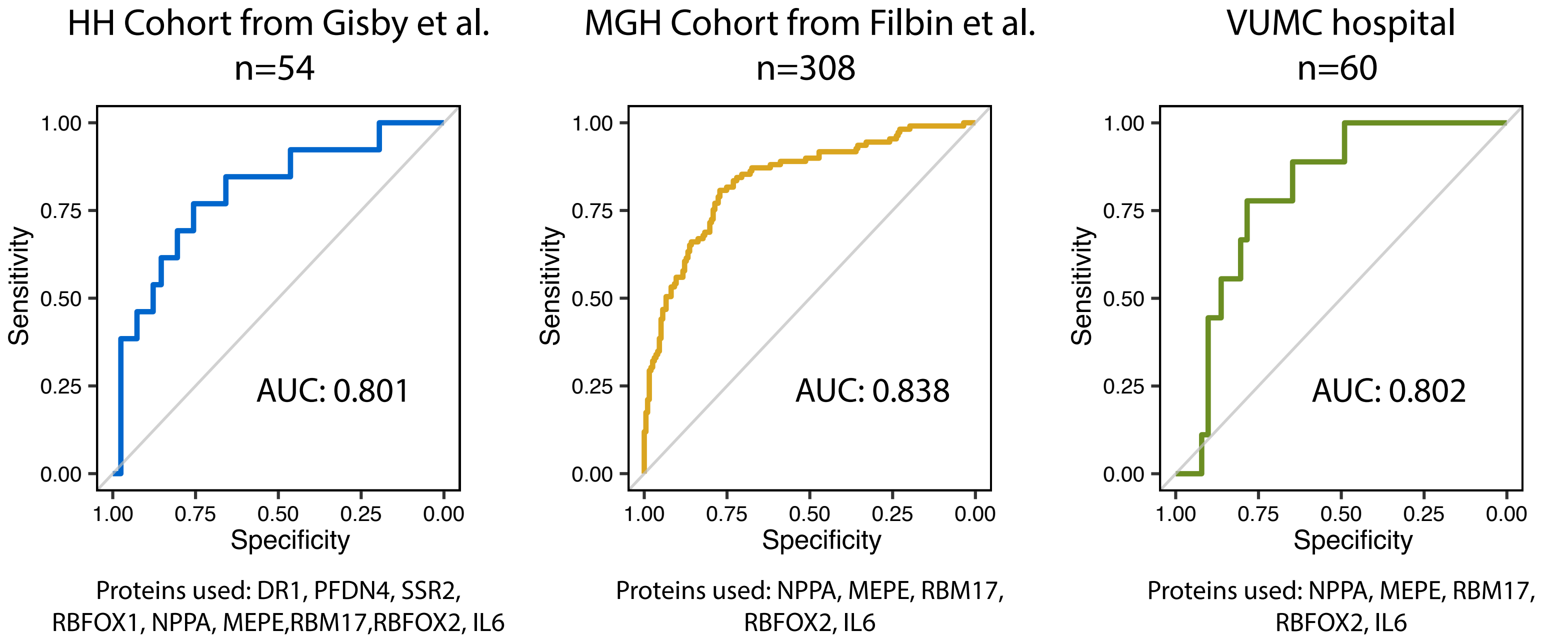

External validation of the Least Absolute Shrinkage and Selection Operator (LASSO) model across two independent study cohorts that employed the Somascan platform. The area under the curve (AUC) is given. HH = Hammersmith Hospital, MGH = Massachusetts General Hospital.VUMC = Amsterdam UMC, location Vrije Universiteit Medical Center. Full protein names are provided in the data dictionary of the Source data file.

Figure S11 Protein abundance in patients randomised to imatinib or placebo at hospital admission

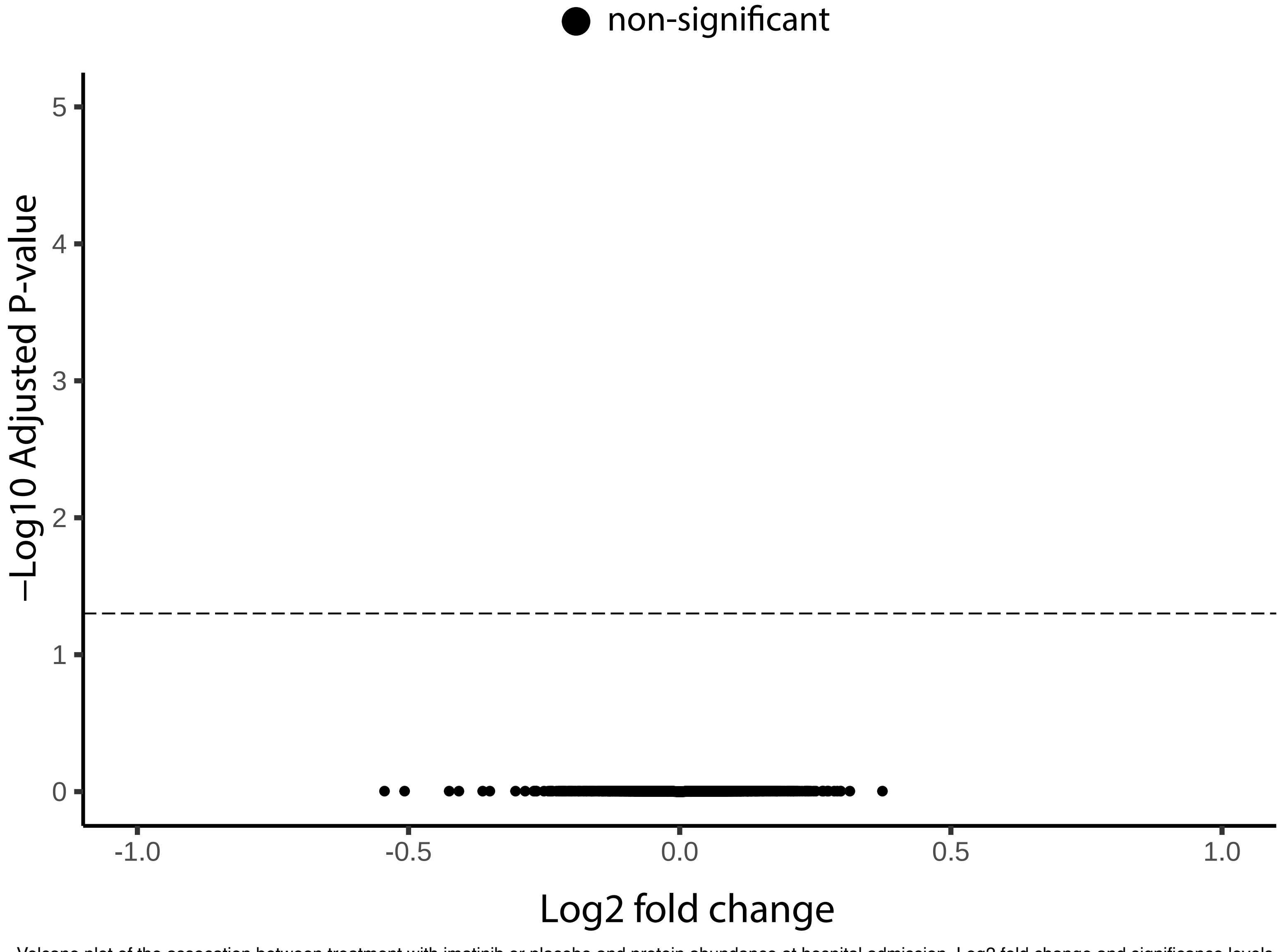

Volcano plot of the assocation between treatment with imatinib or placebo and protein abundance at hospital admission. Log2 fold change and significance levels were calculated using linear mixed models. P-values were adjusted using the Benjamini-Hochberg method. n = 249 samples/individuals

Figure S12 Relative protein abundance in patients treated with imatinib or placebo after 3 days

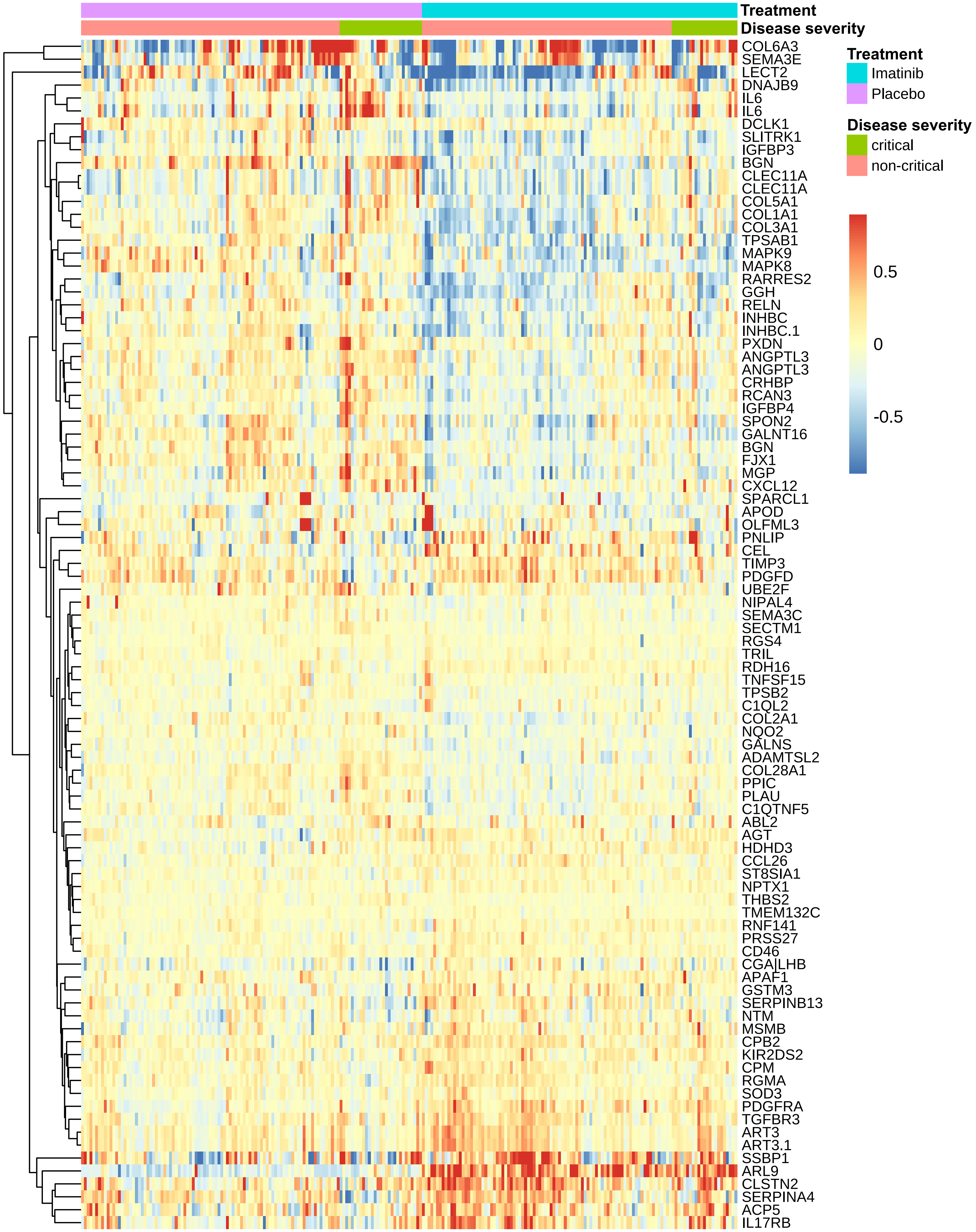

The heatmap was created using the pheatmap function from the pheatmap package on the output from linear mixed models. n= 231 samples/individuals

Figure S13 Effect of imatinib on the transcriptome of Syrian hamster lungs

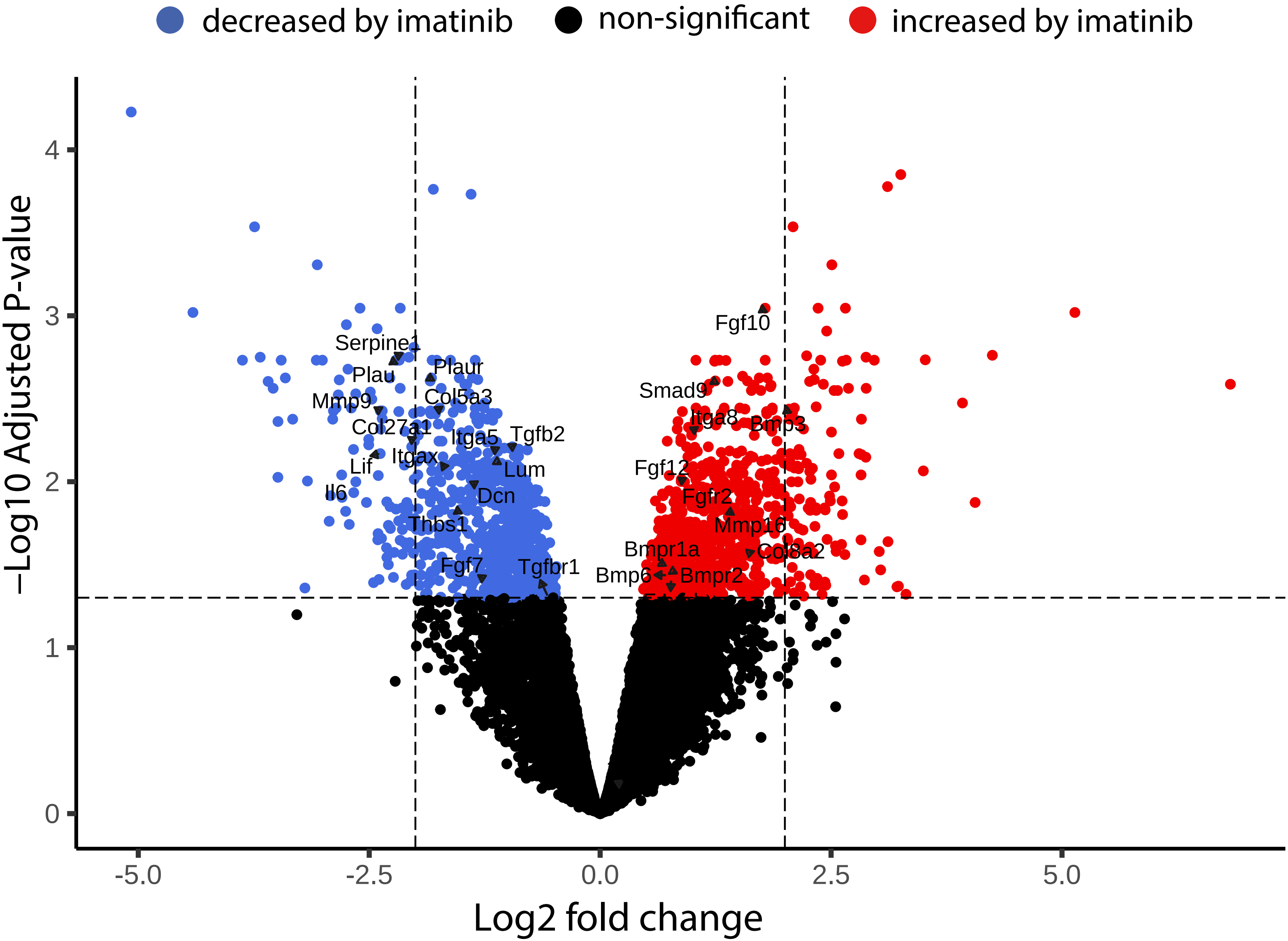

RNA sequencing data was retrieved from an external study and reanalyzed. Negative binomial generalized log-linear models were applied for differential expression using the glmLRT function from the edgeR package. P-values were adjusted using the Benjamini-Hochberg method. n=12 Syrian hamsters *(Mesocricetus auratus)* treated with intragastric imatinib (6) or mock (n=6).

Figure S14 Pathways affected by imatinib in the transcriptome of Syrian hamster lungs

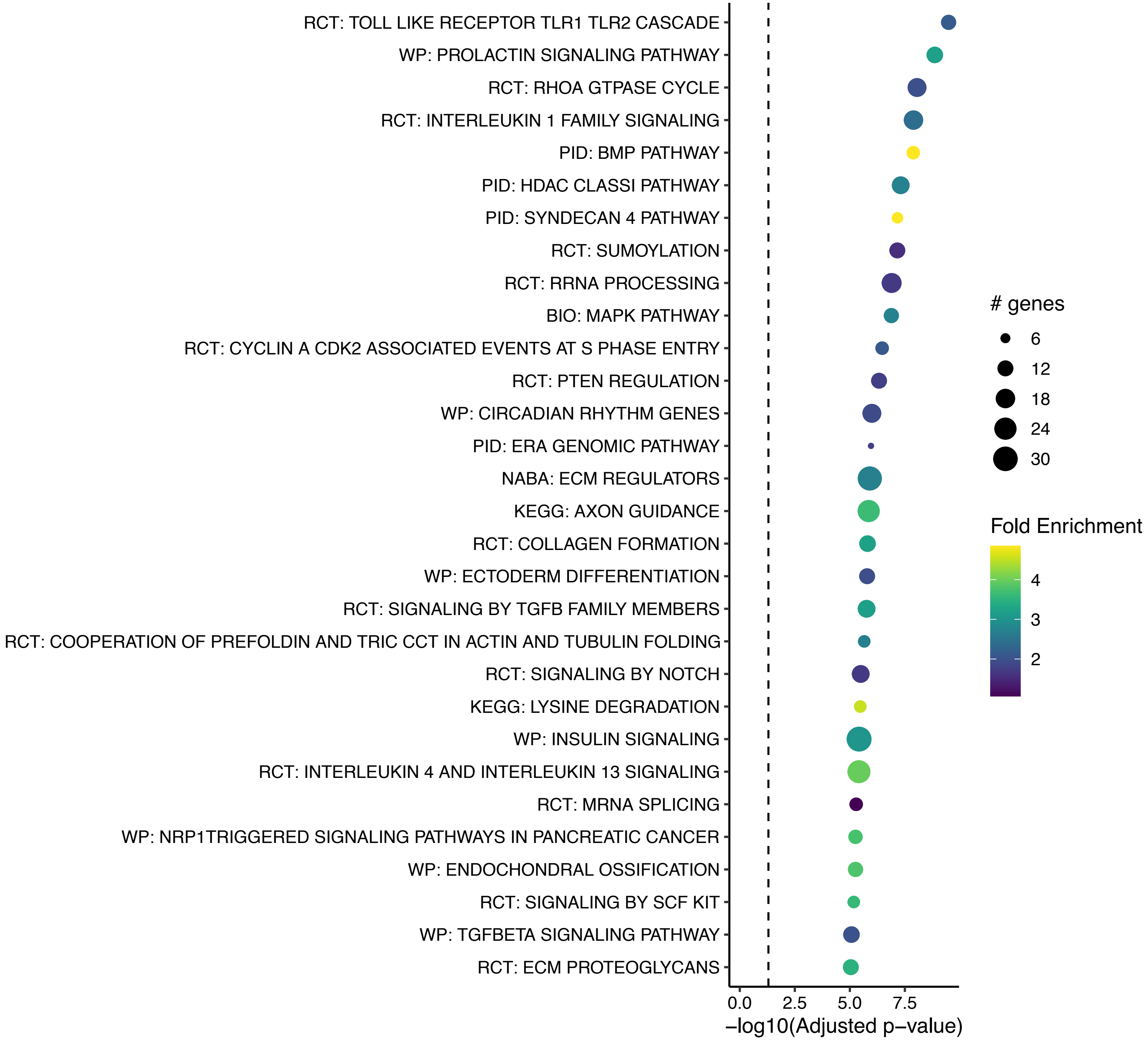

Dotplot of enriched pathways identified by PathfindR. RNA sequencing data was retrieved from an external study and reanalyzed. The 30 most significant clusters are represented by the most significant pathway term of each cluster. Dot size indicates the number of proteins, colors represent fold enrichment. Terms are ordered by significance. BIO = Biocarta, KEGG = Kyoto Encyclopedia of Genes and Genomes, PID = Pathway Interaction Database, RCT = Reactome, WP = Wikipathways. n=12 Syrian hamsters *(Mesocricetus auratus)* treated with intragastric imatinib (6) or mock (n=6).

Figure S15 Genotypic alterations across various cell types in fatal COVID-19

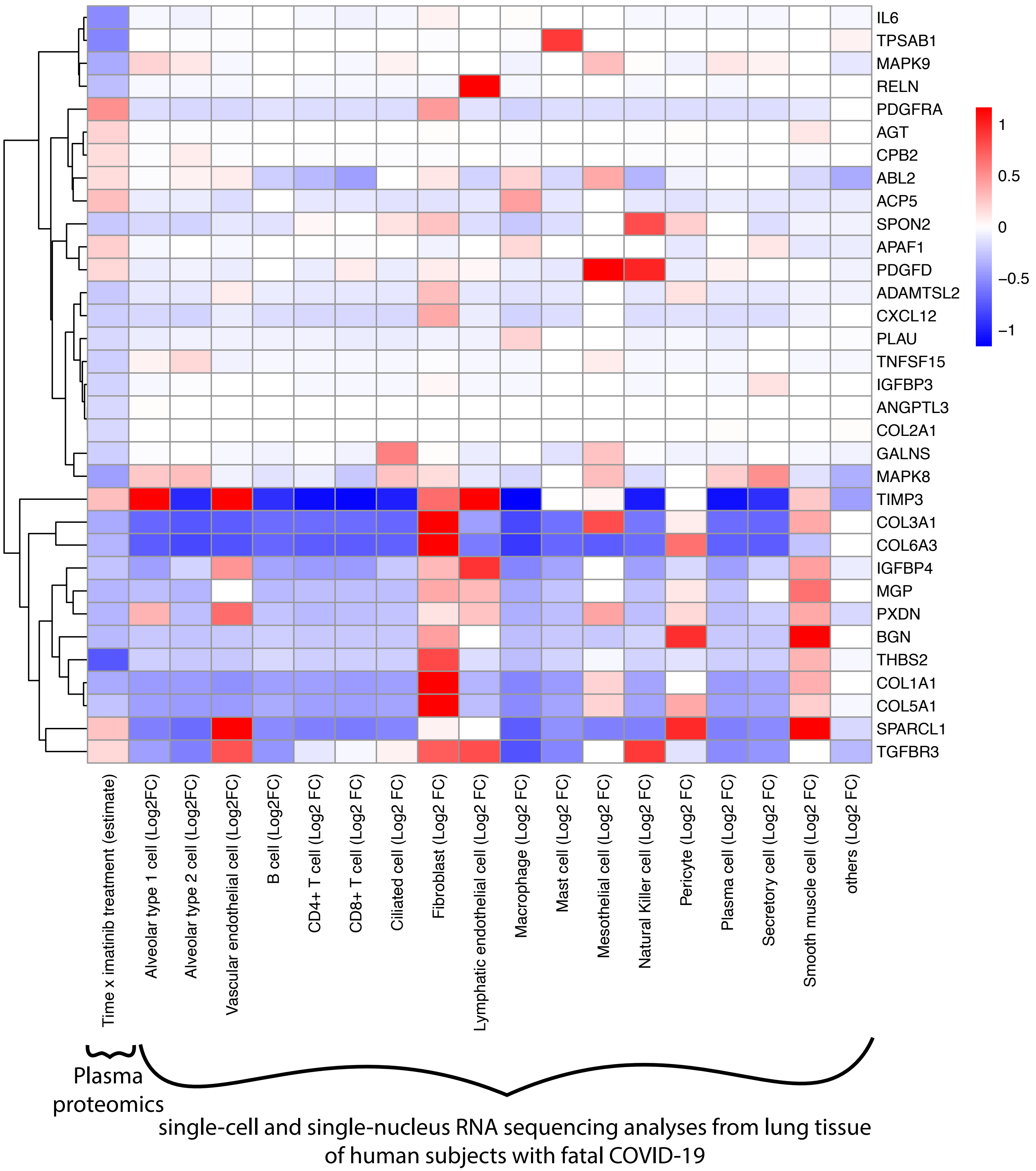

Heatmap of the log2 fold change (FC) across various cell types in lung tissue from human subjects with fatal COVID-19 (n=16 donors; external source) and the estimate of the interaction between time and imatinib treatment in the human plasma proteome derived from linear mixed models (n = 480 samples from 318 individuals). The heatmap was created using the pheatmap function.
