# Supplementary material for: Longitudinal plasma proteomics reveals biomarkers of alveolar-capillary barrier disruption in critically ill COVID-19 patients
Source: Nat Commun. 2024 Jan 25;15:744. doi: 10.1038/s41467-024-44986-w (PMC10811341; doi:10.1038/s41467-024-44986-w)
Supplement: Supplementary file 3 — Description of Additional Supplementary Files [file 41467_2024_44986_MOESM3_ESM.pdf]

Description of Additional Supplementary Files

Supplementary Datasets

Supplementary Data 1. Protein content, full protein names and associated gene and Uniprot identifiers. SeqID = Somalogic sequence identifier, Uniprot ID = Uniprot identifier, GeneID = Gene identifier.

Supplementary Data 2. Differential effects of critical disease at baseline, results from linear mixed models. df= degrees of freedom, GeneID = Gene identifier, SE = Standard Error, SeqID = Somalogic sequence identifier, Uniprot ID = Uniprot identifier.

Supplementary Data 3. Differential effects of critical disease at follow-up, results from linear mixed models. df= degrees of freedom, GeneID = Gene identifier, SE = Standard Error, SeqID = Somalogic sequence identifier, Uniprot ID = Uniprot identifier.

Supplementary Data 4. Differential effects of time*critical disease, results from linear mixed models. df= degrees of freedom, GeneID = Gene identifier, SE = Standard Error, SeqID = Somalogic sequence identifier, Uniprot ID = Uniprot identifier.

Supplementary Data 5. Association of protein expression with time to death/intubation, results from cox proportional hazards regression. coef = coefficient, exp = exponential, se = standard error, SeqID = Somalogic sequence identifier, Uniprot ID = Uniprot identifier.

Supplementary Data 6. Differental effect of imatinib treatment at baseline, results from linear mixed models. df= degrees of freedom, GeneID = Gene identifier, SE = Standard Error, SeqID = Somalogic sequence identifier, Uniprot ID = Uniprot identifier.

Supplementary Data 7. Differential effects of time*imatinib treatment, results from linear mixed models. df= degrees of freedom, GeneID = Gene identifier, SE = Standard Error, SeqID = Somalogic sequence identifier, Uniprot ID = Uniprot identifier.

Supplementary Data 8. Differential effects of imatinib on the hamster transcriptome, results from negative binomial generalized log-linear models. HGNC = Human Genome Organisation (HUGO) Gene Nomenclature Committee, logCPM = logarithmic counts per million, logFC = logarithmic fold change, LR = likelihood ratio.

Supplementary Data 9. Enriched terms for critical disease at baseline, results from pathway analysis. ID = identifier.

Supplementary Data 10. Enriched terms for critical disease at follow-up, results from pathway analysis. ID = identifier.

Supplementary Data 11. Enriched terms for time* critical disease, results from pathway analysis. ID = identifier.

Supplementary Data 12. Enriched terms for time to death/intubation, results from pathway analysis. ID = identifier.

Supplementary Data 13. Enriched terms for time*imatinib treatment, results from pathway analysis. ID = identifier.

Supplementary Data 14. Enriched terms for transcriptome analysis in syrian hamster lungs, results from pathway analysis. ID = identifier.

Supplementary Data 15. Differential effects of fatal COVID-19 on the transcriptome across various cells of human lungs. qval = q-value, log2FC = logarithmic (base 2) fold change, SeqID = Somalogic sequence identifier, Uniprot ID = Uniprot identifier, AT1 = Alveolar type 1, AT1 = Alveolar type 2, NK = Natural Killer.
