# Supplementary material for: Longitudinal plasma proteomics reveals biomarkers of alveolar-capillary barrier disruption in critically ill COVID-19 patients
Source: Nat Commun. 2024 Jan 25;15:744. doi: 10.1038/s41467-024-44986-w (PMC10811341; doi:10.1038/s41467-024-44986-w)
Supplement: Supplementary file 5 — Reporting Summary [file 41467_2024_44986_MOESM5_ESM.pdf]

nature portfolio

Corresponding author(s): Jurjan Aman

Last updated by author(s): Jan 8, 2024

Reporting Summary

Nature Portfolio wishes to improve the reproducibility of the work that we publish. This form provides structure for consistency and transparency in reporting. For further information on Nature Portfolio policies, see our Editorial Policies and the Editorial Policy Checklist.

Statistics

For all statistical analyses, confirm that the following items are present in the figure legend, table legend, main text, or Methods section.

n/a	Confirmed	
☐	☒	The exact sample size (*n*) for each experimental group/condition, given as a discrete number and unit of measurement
☐	☒	A statement on whether measurements were taken from distinct samples or whether the same sample was measured repeatedly
☐	☒	The statistical test(s) used AND whether they are one- or two-sided<br>*Only common tests should be described solely by name; describe more complex techniques in the Methods section.*
☐	☒	A description of all covariates tested
☐	☒	A description of any assumptions or corrections, such as tests of normality and adjustment for multiple comparisons
☐	☒	A full description of the statistical parameters including central tendency (e.g. means) or other basic estimates (e.g. regression coefficient) AND variation (e.g. standard deviation) or associated estimates of uncertainty (e.g. confidence intervals)
☐	☒	For null hypothesis testing, the test statistic (e.g. *F*, *t*, *r*) with confidence intervals, effect sizes, degrees of freedom and *P* value noted<br>*Give P values as exact values whenever suitable.*
☒	☐	For Bayesian analysis, information on the choice of priors and Markov chain Monte Carlo settings
☐	☒	For hierarchical and complex designs, identification of the appropriate level for tests and full reporting of outcomes
☐	☒	Estimates of effect sizes (e.g. Cohen's *d*, Pearson's *r*), indicating how they were calculated

Our web collection on statistics for biologists contains articles on many of the points above.

Software and code

Policy information about availability of computer code

Data collection: Clinical data was collected in Castor EDC; Protein data was delivered by Somalogic Inc

Data analysis: R version 4.2.1 and RStudio version 2023.03.0 Cherry Blossom. Packages used for statistical analysis include pathfindR, lmerTest, caret, edgeR, scater, survival, glmnet,biomaRt,car, ggplot2, survminer, SingleCellExperiment, scuttle,stringr, emmeans, dplyr, StanHeaders, tableone,plyr, formattable, readxl, lme4, EnhancedVolcano, ggrepel, tidyr, M3C, pander and the R stats package. The R script required to reproduce the analyses is available from Zenodo (DOI: https://doi.org/10.5281/zenodo.10142777).

For manuscripts utilizing custom algorithms or software that are central to the research but not yet described in published literature, software must be made available to editors and reviewers. We strongly encourage code deposition in a community repository (e.g. GitHub). See the Nature Portfolio guidelines for submitting code & software for further information.

Data

Policy information about availability of data

All manuscripts must include a data availability statement. This statement should provide the following information, where applicable:

- Accession codes, unique identifiers, or web links for publicly available datasets
- A description of any restrictions on data availability
- For clinical datasets or third party data, please ensure that the statement adheres to our policy

Pseudonymized source data utilized in this study and generated data are publicly available without restriction. The raw data required for deconvolution of RNA-seq analysis in human lung tissue can be obtained from DOI: 10.1038/s41586-021-03570-8.

For this study, external RNA-seq data from SARS-CoV-2 infected hamsters was retrieved from https://doi.org/10.1038/s41423-023-00985-3. SomaScan proteomics data from Filbin et al. 17 can be accessed from DOI: 10.1016/j.xcrm.2021.100287. SomaScan proteomics data from Gisby et al. 18can be accessed from https://doi.org/10.1038/s41467-022-35454-4.

For pathway analysis, a Protein Interaction Network (PIN) was retrieved from Biogrid (version 4.4.224): https://downloads.thebiogrid.org/BioGRID

Protein sets were defined using the Molecular Signatures Database (MSigDB) C2 canonical pathways, which is available through: https://www.gsea-msigdb.org/gsea/msigdb/human/collections.jsp

Research involving human participants, their data, or biological material

Policy information about studies with human participants or human data. See also policy information about sex, gender (identity/presentation), and sexual orientation and race, ethnicity and racism.

Reporting on sex and gender	The study was performed in both males (n=222, 70%) and female (n=96, 30%). Sex refers solely to biological attribute.
Reporting on race, ethnicity, or other socially relevant groupings	No reports were made on race, ethnicity or other socially relevant groupings
Population characteristics	Eligible subjects were above 18 years of age, had a (RT-PCR)- confirmed SARS-CoV-2 infection, and were hypoxemic or required supplemental oxygen to maintain a peripheral oxygen saturation above 94%. A complete overview of population characteristics is provided in Table S1.
Recruitment	Hospitalized atients were recruited Between March 31, 2020, and Jan 4, 2021 from 13 academic or teaching hospitals as stated in: Aman J, Duijvelaar E, Botros L, Kianzad A, Schippers JR, Smeele PJ, et al. Imatinib in patients with severe COVID-19: a randomised, double-blind, placebo-controlled, clinical trial. Lancet Respir Med. 2021;9(9):957–68. For this secondary analysis on the plasma proteome, all patients of whom a plasma sample was available, were included. No significant differences were observed between patients from whom a plasma sample was available versus those without a plasma sample available.
Ethics oversight	This trial was conducted in compliance with all relevant ethical regulations, including the guidelines of the International Conference on Harmonization Good Clinical Practice and the Declaration of Helsinki. The trial protocol was approved by the medical ethics committee of the Amsterdam UMC (VUmc, Amsterdam, Netherlands) and institutional review board of the Amsterdam UMC, location VUmc. Study participants did not receive compensation for their involvement. This study was registered with the EU Clinical Trials Register (EudraCT 2020–001236–10) and Netherlands Trial Register (NL8491).

Note that full information on the approval of the study protocol must also be provided in the manuscript.

Field-specific reporting

Please select the one below that is the best fit for your research. If you are not sure, read the appropriate sections before making your selection.

☒ Life sciences ☐ Behavioural & social sciences ☐ Ecological, evolutionary & environmental sciences

For a reference copy of the document with all sections, see nature.com/documents/nr-reporting-summary-flat.pdf

Life sciences study design

All studies must disclose on these points even when the disclosure is negative.

Sample size	A sample size calculation was performed for the CounterCOVID trial. The data of all patients included in the CounterCOVID trial with at least one plasma sample available during the first three days of treatment were used for this study. An anticipated sample size of 386 patients was calculated based on an anticipated hazard ratio of 1.39 in the primary outcome (time to discontinuation of ventilation and supplemental oxygen for more than 48 consecutive hours, while being alive during a 28-day period after randomization)of the CounterCOVID trial, with an

	alpha 0.025 for superiority of the intervention and a beta 0.20. More details are provided in section 4.4 of the study protocol.
Data exclusions	55 Samples were excluded from data analyses because they did not pass quality control
Replication	Proteomics measurements were only performed once by Somalogic Inc, and were thus not replicated. The predictive value of a nine-protein panel on the development of critical illness was externally validated in two independent study cohorts.
Randomization	Randomisation was done with the Castor Electronic Data Capturing System (Castor EDC; Amsterdam, Netherlands). Randomisation was stratified by hospital site using variable block sizes of two, four, or six patients. All patients, health care providers and study investigators were blinded to treatment allocation.
Blinding	All patients, health care providers and study investigators were blinded to treatment allocation

Reporting for specific materials, systems and methods

We require information from authors about some types of materials, experimental systems and methods used in many studies. Here, indicate whether each material, system or method listed is relevant to your study. If you are not sure if a list item applies to your research, read the appropriate section before selecting a response.

Materials & experimental systems

n/a	Involved in the study
☒	☐ Antibodies
☒	☐ Eukaryotic cell lines
☒	☐ Palaeontology and archaeology
☒	☐ Animals and other organisms
☐	☒ Clinical data
☒	☐ Dual use research of concern
☒	☐ Plants

Methods

n/a	Involved in the study
☒	☐ ChIP-seq
☒	☐ Flow cytometry
☒	☐ MRI-based neuroimaging

Clinical data

Policy information about clinical studies

All manuscripts should comply with the ICMJE guidelines for publication of clinical research and a completed CONSORT checklist must be included with all submissions.

Clinical trial registration	This trial was registered with the EU Clinical Trials Register (EudraCT 2020–001236–10, https://www.clinicaltrialsregister.eu/ctr-search/trial/2020-001236-10/NL) and Netherlands Trial Register (NL8491, https://www.trialregister.nl/trial/8491).
Study protocol	The full study protocol (version 6.1) can be found here: https://doi.org/10.1016/S2213-2600(21)00237-X
Data collection	Between March 31, 2020, and Jan 4, 2021, 385 participants participated in the associated double-blind, placebo-controlled randomised clinical trial. From 318 out of 385 participants, clinical data and proteomic analysis were used for this secondary study. Full details of the primary clinical trial is provided in: Aman J, Duijvelaar E, Botros L, Kianzad A, Schippers JR, Smeele PJ, et al. Imatinib in patients with severe COVID-19: a randomised, double-blind, placebo-controlled, clinical trial. Lancet Respir Med. 2021;9(9):957–68. Briefly, study participants were recruited from 13 participating hospitals in the Netherlands. Eligible participants were above 18 years of age, had a reverse transcription polymerase chain reaction (RT-PCR)- confirmed SARS-CoV-2 infection, and were hypoxemic or required supplemental oxygen to maintain a peripheral oxygen saturation above 94%. The exclusion criteria comprised, but were not limited to, pre-existing severe pulmonary disease, pre-existing heart failure and concomitant treatment with medications known to strongly interact with imatinib. After obtaining written informed consent, participants were 1:1 randomised to receive a loading dose of 800 mg imatinib, followed by 400 mg of imatinib once daily or an equivalent number of placebo tablets for a total of 10 days. Clinical data was collected daily during hospitalization, registered in Castor EDC and monitored by an independent contract research organisation.
Outcomes	A complete overview of predefined study outcomes of the CounterCOVID trial is provided in section 8 of the study protocol. All study endpoints related to this secondary analysis are provided in the methods section. In brief, the main study outcomes comprise the effect of critical illness and/or imatinib treatment on plasma proteome abundance. Critical illness was defined as the need for invasive ventilation and/or fatal outcome during the 90-day follow-up period
